# Comparison of electronic versus conventional assessment methods in ophthalmology residents; a learner assessment scholarship study

**DOI:** 10.1186/s12909-021-02759-9

**Published:** 2021-06-13

**Authors:** Hamidreza Hasani, Mehrnoosh Khoshnoodifar, Armin Khavandegar, Soleyman Ahmadi, Saba Alijani, Aidin Mobedi, Shaghayegh Tarani, Benyamin Vafadar, Ramin Tajbakhsh, Mehdi Rezaei, Soraya Parvari, Sara Shamsoddini, David I. Silbert

**Affiliations:** 1 Eye Research Center, The Five Senses Institute, Rassoul Akram Hospital, Iran University of Medical Sciences, Tehran, Iran; 2 Department of Ophthalmology, Madani Medical Center, School of Medicine, Alborz University of Medical Sciences, Karaj, Iran; 3 School of Management & Medical Education, Shahid Beheshti University of Medical Sciences, Tehran, Iran; 4 Student Research Committee, Alborz University of Medical Sciences, Karaj, Iran; 5 Non-Communicable Disease Research Center, Alborz University of Medical Sciences, Karaj, Iran; 6 Department of Emergency Medicine, School of Medicine, Alborz University of Medical Sciences, Karaj, Iran; 7 Department of Anatomical Sciences, School of Medicine, Alborz University of Medical Sciences, Karaj, Iran; 8 Shams Eye and Skin Infirmary, Tehran, Iran; 9 Conestoga Eye, Lancaster, PA USA

**Keywords:** Electronic, Conventional, Assessment, Ophthalmology residents, Scholarship study

## Abstract

**Background:**

Assessment is a necessary part of training postgraduate medical residents. The implementation of methods located at the “shows how” level of Miller’s pyramid is believed to be more effective than previous conventional tools. In this study, we quantitatively compared electronic and conventional methods in assessing ophthalmology residents.

**Methods:**

In this retrospective study, eight different conventional methods of assessment including residents’ attendance, logbook, scholarship and research skills, journal club, outpatient department participation, Multiple Choice Question (MCQ), Objective Structured Clinical Examination (OSCE), and professionalism/360-degree (as one complex) were used to assess 24 ophthalmology residents of all grades. Electronic media consisting of an online Patient Management Problem (e-PMP), and modified electronic OSCE (me-OSCE) tests performed 3 weeks later were also evaluated for each of the 24 residents. Quantitative analysis was then performed comparing the conventional and electronic assessment tools, statistically assessing the correlation between the two approaches.

**Results:**

Twenty-four ophthalmology residents of different grades were included in this study. In the electronic assessment, average e-PMP scores (48.01 ± 12.40) were much lower than me-OSCE (65.34 ± 17.11). The total average electronic score was 56.67 ± 11.28, while the total average conventional score was 80.74 ± 5.99. Female and male residents’ average scores in the electronic and conventional method were (59.15 ± 12.32 versus 83.01 ± 4.95) and (55.19 ± 10.77 versus 79.38 ± 6.29), respectively. The correlation between modified electronic OSCE and all conventional methods was not statistically significant (*P*-value >0.05). Correlation between e-PMP and six conventional methods, consisting of professionalism/360-degree assessment tool, logbook, research skills, Multiple Choice Questions, Outpatient department participation, and Journal club active participation was statistically significant (P-value < 0.05). The overall correlation between conventional and electronic methods was significant (P-value = 0.017).

**Conclusion:**

In this study, we conclude that electronic PMP can be used alongside all conventional tools, and overall, e-assessment methods could replace currently used conventional methods. Combined electronic PMP and me-OSCE can be used as a replacement for currently used gold-standard assessment methods, including 360-degree assessment.

## Background

In recent decades, many authors have tried to provide a proper definition for “competency” in medical education. Epstein et al. define competency as: “*the habitual and judicious use of communication, knowledge, technical skills, clinical reasoning, emotions, values, and reflection in daily practice for the benefit of the individuals and communities being served.*” [1], but this is rarely what we measure in testing students. Various types of competency evaluation frameworks have evolved over the years, including Accreditation Council for Graduate Medical Education “ACGME” and Brown in the United States, Association of Faculties of Medicine of Canada “AFMC,” and canMEDs in Canada, Scottish doctor, Good medical practice standard in the UK. In every one of these frameworks, different types of competencies exist, including knowledge, skills, professionalism, communication, and empathy [2–4]. Based on the three-circle model presented by Harden, competencies could be categorized into three main groups consisting of the competencies related to “performance of tasks,” competencies related to “approach of students to tasks,” and finally, “professionalism related competencies” [5]. In the competency-based education (CBE) era, each medical school defines specified competencies adjusted to local needs, developing milestones, and clarifying them [6].

An important part of training in every discipline is assessment. Medical students continuously require assessment throughout their medical journey to identify their defects in knowledge and skills, recognize their strengths, and remediate in a non-threatening and productive way [6, 7]. Due to life and death decisions, the assessment of healthcare professionals needs to go beyond paper-based testing. Proper assessment should evaluate judgment, attitude and behavior in addition to knowledge, and skills [6]. All assessment methods are imperfect and have specific flaws and strengths, which can be identified through validity and reliability index calculations [8, 9].

**Fig. 1:**
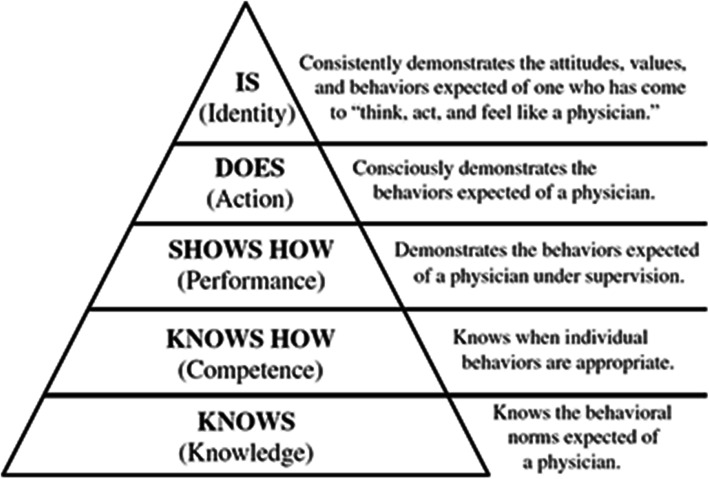
Modified Miller’s pyramid adopted from Miller GE [10] and Cruess RL [11]. Legend: Modified (od amended) Miller’s pyramid presented by Cruess RL. et al. in 2016 after the original pyramid presented by Miller in 1990. A “to be” level is added in the apex of “amended Miller’s pyramid” compared to the original one

In the late twentieth century, Miller presented his pyramid in which assessment was not only limited to knowledge, but also skills, attitude, performance, and professionalism. Miller’s pyramid consists of 4 sequential levels of assessment beginning at the “know” level followed by “knows how” and “shows how” and ended at the “does” level [10]. In 2016 Cruess et al. made new amendments to Miller Pyramid, which eventually resulted in an “is” level at the apex [11]. Modified Miller’s pyramid is depicted in Fig. 1.

Assessment methods located at a higher level are more validated and have more impact than those at the lower levels [10, 12]. The low levels of the Miller Pyramid do not provide a reasonable estimate of clinical reasoning [13]. Objective Structured Clinical Examination (OSCE) and Patient Management Problem (PMP) are two assessment methods at the “shows how” level used in this article. These assessment methods are based and students’ decision-making in simulated conditions with simulated patients [14]..

Considering the importance of OSCE and PMP in the assessment of medical trainees and confronting a lack of studies investigating the efficacy of these two assessment tools simultaneously, we decided to apply modified electronic OSCE and PMP assessment methods and make a quantitative comparison with conventional tools in the evaluation of ophthalmology residents.

## Methods

We designed this retrospective study in two stages evaluating primary conventional and then electronic assessment methods. The electronic method consisted of me-OSCE and e-PMP questions, and conventional methods included eight different components. OSCE was implemented in both electronic and conventional methods to provide greater validity and reliability for both methods. Twenty-four ophthalmology residents, 9 females and 15 males, participated at all conventional exam stages and eventually, 3 weeks later in the electronic assessment.

In electronic OSCE, clinical examinations were performed on patients (real or simulated) in different clinical stations. Images and videos were presented electronically on computers. Examples of clinical stations included refractions on manikins, keratometry, and lensometry on real and simulated patients. Likewise, the application of real patients and simulated patients was performed in conventional OSCE whenever possible.

We use the term “Modified Electronic Objective Structured clinical examination” abbreviated as “me-OSCE” for the electronic type of OSCE. Ophthalmology para-clinics content used in me-OSCE were topography, tomography tests (Orb Scan, Pentacam, Sirius), perimetry (Humphrey visual field), ocular sonography (A&B scan), OCT, fluorescein angiography, (Ocular Coherence Tomography), AS-OCT (Anterior Segment OCT) and other tests provided by an expert panel of ophthalmology attending physicians.

### Conventional methods

Preliminary conventional assessment methods were implemented 3 weeks before final electronic examinations. Preliminary conventional assessment methods consisted of eight different parts: residents’ attendance, logbook, scholarship and research skills, journal club, outpatient department participation, MCQ, OSCE, and 360 degrees/professionalism (as one complex);

Residents’ attendance was a fundamental tool for assessing the active participation of residents in morning reports, grand rounds, workshops, undergraduate training, and scientific discussion. Daily Logbooks in either physical or electronic daily recording of the performed procedure, observed patients, and learning experiences were reviewed. We used the physical logbook as a formative assessment tool at the “does” level of Miller’s pyramid.

Outpatient department (OPD), observership training, and other educational locations (hospital, emergency ward) were used along with journal club activity as a measurable tool for assessment. MCQ was previously the most commonly used assessment tool in many fields of medicine both in undergraduate and postgraduate training, even in continuing medical education examination assessing knowledge in “knows” level of Miller’s pyramid [15]. Furthermore, other evaluation types consisted of logbooks, research skills, OSCE, and eventually, 360-degree were used. A crucial part of our 360-degree assessment evaluated separately and specifically was professionalism.

In logbooks, the residents’ activity in the emergency ward, including the number of visited patients, types of performed procedures and treatments, and types of visited patients, were considered. Resident’s operating room activities, including types of surgeries, probable adverse effects of surgery, and patients’ follow-up, were considered. Other parameters that were taken into consideration in the logbook were stated activities in clinics and hospital wards. Furthermore, logbooks were used to facilitate research skills’ assessments. Residents were asked to fill research parts in logbooks, including types of presented patients in journal club and summary of presentations.

### Electronic PMP (e-PMP) and modified electronic OSCE (me-OSCE); presentation of a new assessment method

Scenarios were modified for better adjustment to OSCE, and simulated patients were trained for casting their role in 11 stationary questions. Trial simulated patient role casting for OSCE was held to identify flaws. Separate explanatory workshops for ophthalmology residents and faculty members were held, and the feedback was gathered from the students and faculty members separately and analyzed to identify strengths and weaknesses. A training workshop was held to acquaint residents with the rules and instructions.

### Developing questions

PMP questions included the main body consisting of a real scenario, drug history, past medical history, past ocular surgeries, biomicroscopic slit-lamp examination, and current intraocular pressure.

In me-OSCE, each resident was provided a specific monitor. In each monitor, there were electronic stations of question. With each station, there were three specific parts. Initially, the main text presenting the patient’s condition was shown, then, specific paraclinical images related to the patient’s condition were presented.

Finally, the video of the real patient’s condition, made from real patient vignettes, was presented. The resident was given a specific amount of time for each electronic station to play the video and observe the paraclinical images. Me-OSCE was used beside PMP to increase the electronic part of the assessment’s reliability, as a recommendation of the expert panel.

### Precise scoring system

Assessment committee approved scores were allocated for each question. The scoring range on each page varied from − 12 to + 15. Summation of the scores for each page would negate whether all answers were chosen by chance to eliminate any chance of getting a score by chance. Considering previous PMP tests when a negative score in each pack could extend to the next pack, the assessment committee set regulations by which the final negative score in each pack should be considered zero. For example, if the examinee score achieved in the first and second pack were − 5 and + 7, respectively, the total score would be + 7, not + 2.

Here, we describe the General scoring system criteria for all questions below; Obviously, all scoring was specified one by one for each question:

- Negative Five points for life-threatening option
- Negative Three points for ineffective, Time-wasting, and harmful option
- Negative one point for ineffective, Time-wasting but not harmful option
- Zero points for neither helpful nor harmful option
- Positive one point for helpful, routine modalities option
- Positive three points for essential option
- Positive five points for absolutely essential option

### Feedback

Feedback exists for each option in PMP questions; In other words, by choosing each option, whether right or wrong, the examinee gets an explanation. This process is called “feedback” and eventually results in an inability to get to the correct option. As an example, if the right option for the first part of a question was OCT was not chosen by the examinee, he or she would not have enough information to answer the rest of the question thoroughly. Final question screening and scoring system development was accomplished with an ophthalmology assessment committee including, faculties in every sub-specialties.

### Choice selection

Questions were categorized into “limited answers” and “unlimited answers.” In “unlimited answers” questions, the examinee could choose one or more options concurrently, while in “limited answers” questions, other options would get deactivated when absolute numbers of options were selected. In both types of questions, by the time the next questions appear, previous questions become observable but not changeable.

### Other tips considered for proper assessment implementation

A pilot trial assessment was performed before the final assessment to diminish any possibility of errors. Participating in a trial assessment was mandatory for all residents. User, software, and hardware information access were provided for residents to get familiar with the environment and specific circumstances. Assessors responsible for question design were not allowed to use scenarios presented in books or morning reports.

### Comparability and validation

As there was no previous study to validate the parameters for comparability, we set up an “expert panel” consisting of faculty and professors from every five sub-specialties. All of them were asked to check each electronic and conventional assessment component and finally to approve it. In other words, the “expert panel” was used as a primary tool for validation. To design high quality-questions in both electronic and conventional parts, the approved ophthalmologic references, including AAO publications, were used. Eventually, all scores were presented on a scale of 100 to enable comparability.

As the exam was the same for all grade residents, the “Expert panel” decided to put a unique difficulty-level coefficient for each type of assessment. For example, the expert panel decided to put a difficulty level coefficient of 1.5 for a total score of first-year residents while the coefficient was 1 for the fourth-year residents. The coefficient varied from one assessment tool to another based on the expert panel’s decision.

### Statistical analysis

Statistical Analyses were performed by IBM SPSS software version 24. A *P*-value of less than 0.05 was considered statistically significant. Categorical data are presented as percent, and Continuous demographic variables are presented as Mean ± Standard deviation or Median (Interquartile range). The t-test was used for the comparison among quantitative data, and in case of abnormal distributions Mann-Whitney U test was used. The correlation between quantitative data was checked through the Pearson correlation test and Spearman rank correlation.

## Results

All scores for all parts of conventional and electronic assessments were converted to a scale of 100. All scores followed a normal distribution pattern. The total electronic average score is the average of all scores achieved in e-PMP and me-OSCE. The total average conventional score was calculated through average scores achieved in eight different parts consisting of residents’ attendance, logbook, scholarship and research skills, journal club participation, outpatient department participation, MCQ, OSCE, and finally professionalism/360 degrees’ assessment; professionalism assessment and 360-degree are an inseparable part of each other; hence the total mark in this part was defined as professionalism/360-degree assessment.

With a total number of 24 residents, 33.3% of participants were first-year residents, 16.7% the second year, 25% third-year, and 25% fourth-year residents. The average score in the electronic part of our assessment, through e-PMP (48.01 ± 12.40), was much lower as compared to the me-OSCE (65.34 ± 17.11). The highest average score was allocated to residents’ attendance (99.79 ± 1.02), while the lowest average score was in OSCE (52.79 ± 12.04). Other assessment tools lie in between; professionalism/360 degree (91.70 ± 4.14), logbook (91.66 ± 10.07), journal club participation (91.1 ± 12.47), OPD participation (90.00 ± 11.32), research skills (79.16 ± 13.80) and finally MCQ (with an average score of 60.10 ± 13.57).

**Fig. 2:**
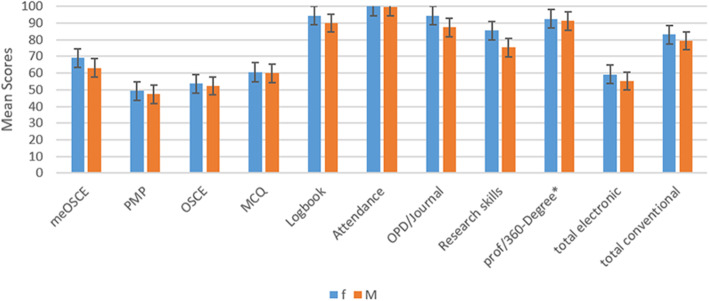
Mean scores achieved through Assessment tools by residents; *Professionalism/ 360-degree. Legend: Mean achieved scores by all residents is seven conventional, two electronic, and total electronic and conventional assessment methods. As demonstrated, female residents’ scores higher in every one of the assessment tools

The total average electronic score was 56.67 ± 11.28, while the total average conventional score was 80.74 ± 5.99. Female residents’ average score was higher in each part of the conventional and electronic assessment and, total score. Female residents’ average score was 59.15 ± 12.32 in electronic and 83.01 ± 4.95 in conventional methods, while male residents’ achieved 79.38 ± 6.29 scores in electronic and 55.19 ± 10.77 scores in conventional methods. The average score achieved through each assessment tool is demonstrated in Fig. 2**.**

The total average score in electronic and conventional tools, respectively, was 50.91 ± 8.94 and 76.15 ± 2.83 in the first grades, 58.12 ± 5.34 and 83.14 ± 1.86 in second grades, 67.08 ± 6.81 and 81.00 ± 7.87 in third grades, and 53.00 ± 14.46 and 85.00 ± 5.44 in last-grade residents.

There was a significant correlation between electronic PMP and all parts of conventional methods except attendance. (*P*-values are demonstrated in Table 1.) In other words, PMP could be taken into account as a supplementary method besides all conventional assessment tools except one related to the attendance of residents (P-value = 0.253, Pearson = − 0.243).

**Table 1:** Correlation between all assessment methods and electronic assessment tools and gold-standard assessment

		me-OSCE	e-PMP	attendance	Prof/ 360^a^	Logbook	Journal Club participation	OPD ^b^	Research skills	MCQ	OSCE
me-OSCE	Correlation *P*-value	1	0.147 0.494	0.378 0.069	−0.008 0.971	0.021 0.923	0.321 0.278	0.241 0.256	−0.001 0.996	0.033 0.877	0.332 0.112
e-PMP	Correlation P-value	0.147 0.494	1	−0.243 0.253	0.499 0.013	−0.519 0.009	0.548 0.033	0.492 0.015	0.528 0.008	0.669 0.000	0.625 0.001
Prof/360^a^	Correlation P-value	−0.008 0.971	0.499 0.013	−0.184 0.391	1	−0.134 0.531	0.815 0.003	0.753 0.000	0.559 0.004	0.341 0.103	0.486 0.016

^a^shortened of professionalism/ 360-degree assessment method. ^b^shortened of Outpatient active participation method. Underlined numbers are significant (*P*-value < 0.05)

Correlation between all assessment methods and electronic assessment tools; due to Due to extensive, repetitive, and precise evaluation applied in professionalism/360-degree’s assessment, it was considered as “gold standard”; Eventually, for comparison of results with the gold standard assessment tool, a correlation was calculated between gold standard assessment tool and all other methods. Underlined numbers present statistically significant correlations

**Fig. 3:**
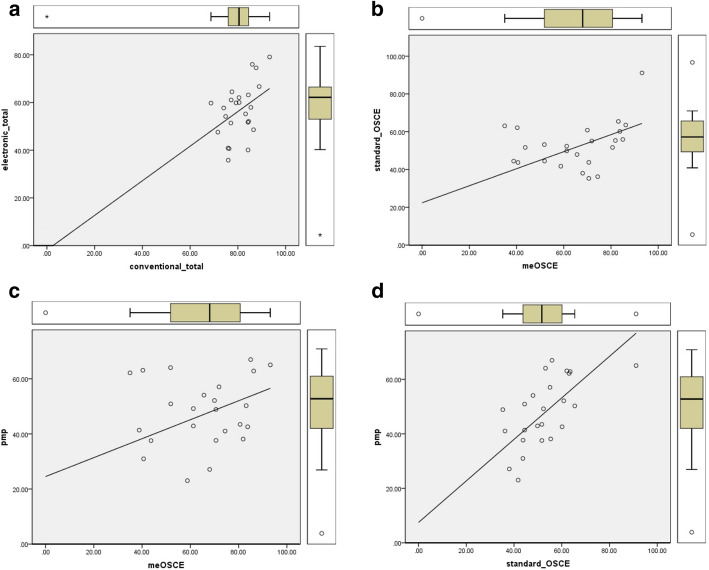
Scatter-plot indicating the nature of data and its distribution. Legend: scatter-plot indicating the nature of data and its distribution and the outliers is demonstrated. **a** The X-axis is the total conventional score and the Y-axis is the total electronic score. **b** The X-axis is the me-OSCE score and the Y-axis is the standard-OSCE score. **c** The X-axis is the me-OSCE score and the Y-axis is the e-PMP score. **d** The X-axis is the standard-OSCE score and the Y-axis is the e-PMP score

**Fig. 4:**
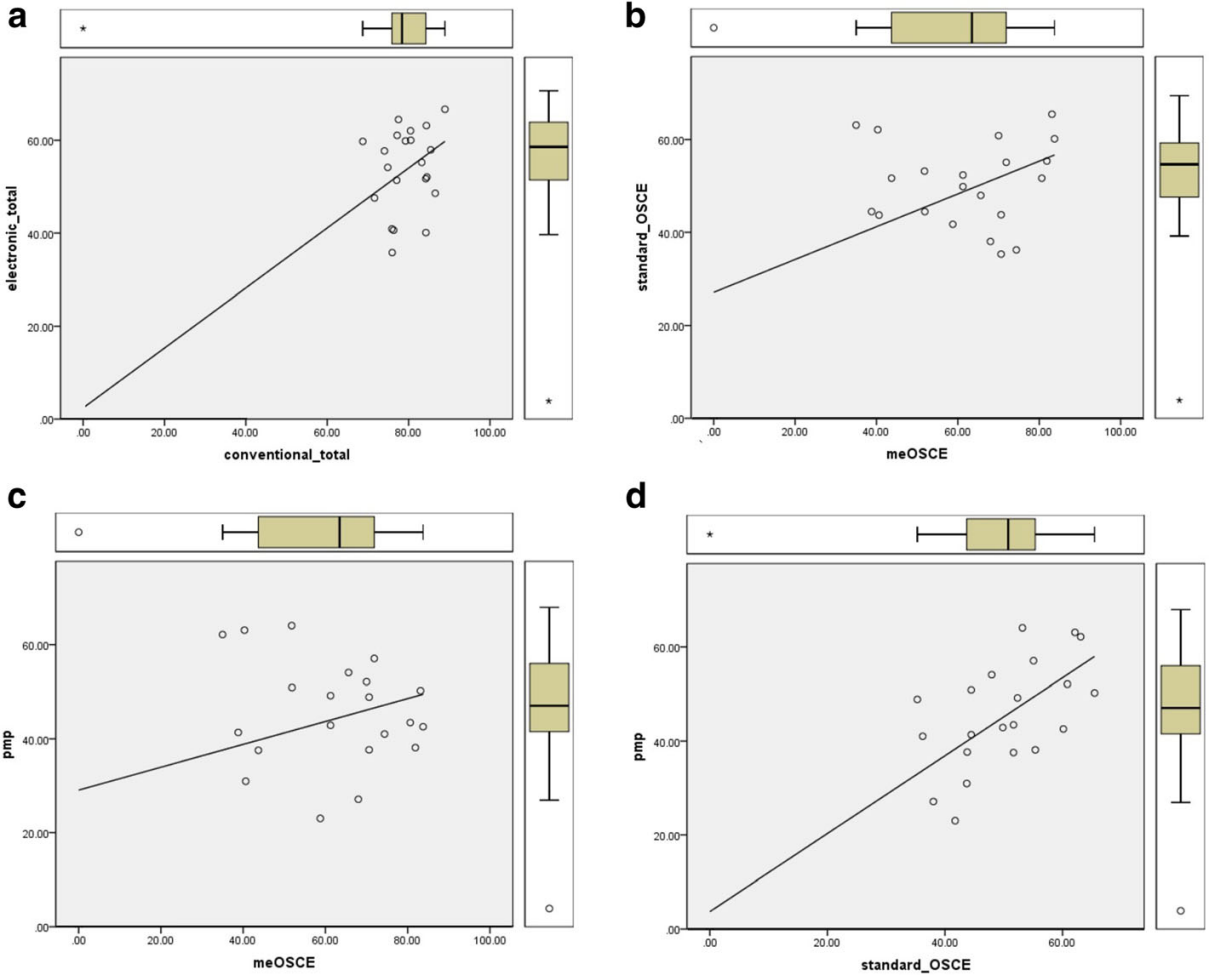
Scatter-plot indicating the nature of data and its distribution, while outliers are removed. Legend: scatter-plot indicating the nature of data and its distribution and the outliers are demonstrated, while outliers are removed. **a** The X-axis is the total conventional score and the Y-axis is the total electronic score. **b** The X-axis is the me-OSCE score and the Y-axis is the standard-OSCE score. **c** The X-axis is the me-OSCE score and the Y-axis is the e-PMP score. **d** The X-axis is the standard-OSCE score and the Y-axis is the e-PMP score

Results of correlations between assessment methods, using Pearson-coefficient, are demonstrated in Table 1. Due to extensive, repetitive, and precise evaluation applied in professionalism/360-degree’s assessment, we defined it as a gold standard tool to be compared. A significant correlation is shown in Table 1. A scatter-plot indicating the nature of data and its distribution is demonstrated in Figure 3**.** To exclude the effect of outliers, we have provided Fig. 4 demonstrating a scatter plot while removing the outliers.

Considering the fact that the top three candidates in most of the assessment methods were believed to be outliers, we performed another analysis to check on the correlations, while removing the outliers. In almost all of the correlations, there were no differences compared to the previous analysis with outliers. Besides, we have provided data distribution and its nature as Fig. 4**.** while removing the outliers.

## Discussion

Learner’s assessment consists of knowledge, skills, attitude, and decision-making authority [6]. To be graduated as a qualified ophthalmologist at the end of a residency-training course, like many other specialties, the acquisition of different types of competency is required. A key element to encourage competency acquirement by students and residents is to launch an “assessment for learning” instead of an “assessment of learning” strategy [7].

The higher level of assessment in Miller’s pyramid model results in the higher achievement of the “assessment for learning” strategy due to the enthusiastic participation of students in their assessment. Moreover, Assessment tools at higher levels of Miller’s pyramid, including OSCE and PMP, are much more integrated into the curriculum [7].

OSCE is well-known to be able to answer the needs of assessors for appropriate evaluation of trainees in almost every field of medicine and even other disciplines, including pharmacy, pharmacology, and psychology [16–19]. In the field of ophthalmology, unlike many other specialties, simulation for OSCE is not feasible in many cases. For example, patients cannot feign keratitis or diabetic retinopathy; although the application of three-dimensional and virtual reality models is expanding in some countries. PMP was considered an applicable approach for clinical skills evaluation improvement in clinical reasoning [20]. A study in Brazil indicated that structures like PMP and OSCE could lead to cognitive and psychomotor skills improvement [21].

We applied MCQ as an essential tool considering residents’ knowledge. Objective Structured Clinical Examination (OSCE) assesses trainees at the “shows how” level of Miller’s Pyramid [22]. Studies evaluating the effect of OSCE in ophthalmology are scarce but seem useful in assessing the required skills and abilities in ophthalmology [23]. Logbook seems to be a useful option for accomplishing specified items in a limited duration [24]. Scholarship skills were previously considered a measurable competency in many popular competency frameworks, including the CARE model [25]. We considered scholarship skills as an inseparable part of the ophthalmology residency and included them in our evaluation.

360-degree feedback is an appropriate tool for assessing communication and interpersonal skills, consisting of feedback to residents, including faculties, residents, patients, and other staff involved in the hospital [26, 27]. Many studies introduced 360-degree as a valid and reliable tool for clinical skills evaluation [28–30]. Likewise, we applied 360-degree based on comments provided by faculties, nurses, operations ward staff, residents, and medical students in the hospital. At the innermost part of Harden’s three-circle model [5], professionalism is meant to motivate trainees for the acquisition of knowledge through independent work [31]. Professionalism, especially in residency, claimed to be the final answer to the needs of society [32].

Confronting the lack of appropriate assessment methods, we implemented e-PMP and me-OSCE alongside previous conventional tools. Finally, we made a quantitative analysis to compare electronic methods with conventional tools. For the implementation of scholarly projects, many criteria have been developed [33]. After a literature review and sessions of discussion, we decided to implement the six standard criteria of Glassick [34].

Proper contextual and environmental preparation is needed for the appropriate implementation of me-OSCE and e-PMP. According to the second Glassick’s criteria, the literature review was performed to anticipate upcoming flaws. Adequate resources were provided, and related workshops were held to acquaint faculty members and residents with instructions and regulations. Training programs for faculty, simulated patients, and residents were conducted.

For appropriate method development based on third Glassick’s criteria, the existence of specific feedback for each question in e-PMP was the turning point of our study among all developed methods previously explained. A controversy evolved whether to provide enough information for examinees who did not correctly answer the previous part of the question. For the first time in ophthalmology residents, we developed me-OSCE and e-PMP alongside conventional methods.

Along with the implementation of effective presentation as fifth Glassick’s criteria, after performing the examination, a report was presented, and the results were announced. Even criticism from faculties of other medical schools was put into account. All suggestions were considered and finally approved if the majority agreed, and the foundation existed.

Following the implementation of me-OSCE and e-PMP, two fourth-year residents achieved top ten ranks in the national board examination; They admitted the electronic assessment method played a crucial role in their achievement (sixth Glassick’s criteria).

In this study, a significant correlation exists between the grade of residents between conventional and electronic assessment. (Pearson = 0.535, *P*-value = 0.007). Correlation between grades of residents and electronic assessment tool was not significant (Pearson = 0.205, *P*-value = 0.338). Adaptation of senior residents to conventional assessment, vast and integrated knowledge, and possessing more clinical skills are crucial factors to get higher scores in conventional tools compared to the junior residents. The third-year residents’ electronic scores were higher than second-year, and the second-year residents’ e-scores were higher than the first years’, but scores achieved by last-year residents in electronic exams were less than scores even achieved by second-year residents.

Higher average scores achieved by female residents’ in each grade both in conventional and electronic assessment methods and even in each assessment method is a notable outcome of this study. Further studies are needed to evaluate intervening factors in the learning abilities of each gender.

Correlation between e-PMP and Logbook is surprisingly negative (Pearson = − 0.519) and significant (*P*-value = 0.009). Investigating the underlying reason, we found out most residents and even faculties assessing Logbooks did not understand its value and neglected them. Finally, we decided to hold related workshops to re-introduce faculties and residents with Log-book and its precious values.

None of the correlations that exist between me-OSCE and the conventional assessment method was significant. As the Pearson correlation index between total electronic score and the total conventional score was 0.481 and still significant (P-value = 0.017), hence, the electronic part can be used as a replacement for the conventional part; even if each part of electronic, me-OSCE as an example, does not have a significant correlation with conventional counterparts.

In this study, due to the multi-target assessment inherent of professionalism/360-degree, it was considered as the gold standard method. Communication and interpersonal skills, among the other dimensions, were evaluated in a considerable period of time in this method from various resources, including faculties, residents, patients, and other staff involved in the hospital.

A combination of a complementary tool (e-PMP) and a substitutional method (me-OSCE), as a total electronic assessment, seems to be an appropriate replacement for a gold-standard professionalism/360-degree assessment tool. Despite providing different figures and statistical analyses, our sample size was not large enough to generalize the results in many ways. Further studies are needed to evaluate the same methods in larger populations.

## Conclusion

Combined electronic PMP and me-OSCE are considered an appropriate replacement for currently used gold-standard assessment methods, including 360-degree assessment.

## Data Availability

The datasets used and analyzed during the current study available from the corresponding author on reasonable request.

## Abbreviations

OSCE: Objective-Structured Clinical Examination
PMP: Patient Management Problem
EMQ: Extended Matching Question
e-PMP: electronic PMP
me-OSCE: modified electronic OSCE
